# Supplementary material for: Modeling glioblastoma heterogeneity as a dynamic network of cell states
Source: Mol Syst Biol. 2021 Sep 16;17(9):e10105. doi: 10.15252/msb.202010105 (PMC8444284; doi:10.15252/msb.202010105)
Supplement: Supplementary file 6 — Source Data for Figure 5 [file MSB-17-e10105-s004.zip › Figure5A_sourcedata/GSEA_3017/nosigdb_stateD.GseaPreranked.1622795010397/RICHARDS_NOWAKOWSKI_SCIENCE_2017_NIN3_UPREG.html]

Details for gene set RICHARDS\_NOWAKOWSKI\_SCIENCE\_2017\_NIN3\_UPREG[GSEA]

|  || Dataset | state23017 |
| Phenotype | NoPhenotypeAvailable |
| Upregulated in class | na\_pos |
| GeneSet | RICHARDS\_NOWAKOWSKI\_SCIENCE\_2017\_NIN3\_UPREG |
| Enrichment Score (ES) | 0.12654625 |
| Normalized Enrichment Score (NES) | 0.7996188 |
| Nominal p-value | 0.75838923 |
| FDR q-value | 0.807917 |
| FWER p-Value | 0.998 |
Table: GSEA Results Summary

  

Fig 1: Enrichment plot: RICHARDS\_NOWAKOWSKI\_SCIENCE\_2017\_NIN3\_UPREG      
 Profile of the Running ES Score & Positions of GeneSet Members on the Rank Ordered List

  

| PROBE | GENE SYMBOL | GENE\_TITLE | RANK IN GENE LIST | RANK METRIC SCORE | RUNNING ES | CORE ENRICHMENT || 1 | SOX11 |  |  | 6 | 0.578 | 0.0603 | Yes |
| 2 | HSP90AB1 |  |  | 32 | 0.393 | 0.0852 | Yes |
| 3 | HSPB1 |  |  | 52 | 0.347 | 0.1095 | Yes |
| 4 | SOX4 |  |  | 91 | 0.298 | 0.1138 | Yes |
| 5 | CHD7 |  |  | 116 | 0.278 | 0.1265 | Yes |
| 6 | PLXNA4 |  |  | 182 | -0.252 | 0.1048 | No |
| 7 | MAPK10 |  |  | 272 | -0.261 | 0.0655 | No |
| 8 | ZC3H14 |  |  | 304 | -0.264 | 0.0712 | No |
| 9 | DLX1 |  |  | 490 | -0.284 | -0.0396 | No |
| 10 | FBXW7 |  |  | 494 | -0.284 | -0.0100 | No |
| 11 | USP9Y |  |  | 516 | -0.287 | 0.0061 | No |
| 12 | NEDD4L |  |  | 595 | -0.299 | -0.0205 | No |
| 13 | ACTR10 |  |  | 615 | -0.303 | -0.0011 | No |
| 14 | SNTG1 |  |  | 697 | -0.319 | -0.0277 | No |
| 15 | DPYSL3 |  |  | 722 | -0.323 | -0.0099 | No |
| 16 | ST8SIA5 |  |  | 737 | -0.327 | 0.0161 | No |
| 17 | RND3 |  |  | 853 | -0.358 | -0.0323 | No |
| 18 | PDE4DIP |  |  | 866 | -0.362 | -0.0008 | No |
| 19 | DST |  |  | 913 | -0.379 | 0.0063 | No |
| 20 | CENPC |  |  | 945 | -0.391 | 0.0264 | No |
| 21 | MEG3 |  |  | 975 | -0.404 | 0.0494 | No |
| 22 | DCLK2 |  |  | 986 | -0.408 | 0.0876 | No |
| 23 | GRIA4 |  |  | 1238 | -0.669 | -0.0308 | No |
| 24 | SYNE2 |  |  | 1282 | -0.829 | 0.0293 | No |
Table: GSEA details [plain text format]

  

Fig 2: RICHARDS\_NOWAKOWSKI\_SCIENCE\_2017\_NIN3\_UPREG: Random ES distribution      
 Gene set null distribution of ES for **RICHARDS\_NOWAKOWSKI\_SCIENCE\_2017\_NIN3\_UPREG**
